# Modeling residents’ multidimensional social capital in China’s neighborhood renewal projects: SEM and MIMIC approaches

**DOI:** 10.3389/fpsyg.2023.1127510

**Published:** 2023-02-10

**Authors:** Ruopeng Huang, Fangyun Xie, Xinyue Fu, Wenli Liu

**Affiliations:** ^1^Management in the Built Environment, Faculty of Architecture and the Built Environment, Delft University of Technology, Delft, Netherlands; ^2^School of Management Science and Real Estate, Chongqing University, Chongqing, China; ^3^Party School of the Chongqing Committee, Chongqing, China; ^4^Department of Civil Engineering, The University of Hong Kong, Pokfulam, Hong Kong SAR, China

**Keywords:** neighborhood renewal, social capital, structural equation modeling, multiple indicators multiple causes, residents behavior

## Abstract

Neighborhood renewal is now an important approach to sustainable urban development in China. However, neighborhood renewal projects are often beset with social problems such as noncooperation from residents, which can be attributed to diverse interests and complex relationships among residents. However, there is little research on resident relations in China and intra-resident conflict. Based on social capital, this study provided a better understanding of resident relationships in neighborhood renewal in China. To this end, we developed a theoretical framework of residents’ multidimensional social capital (structural, relational, and cognitive). Then, a survey was conducted to collect data from 590 residents across China who were experiencing or had experienced neighborhood renewal. Structural equation modeling (SEM) and multiple indicators multiple causes (MIMIC) modeling were used. The results revealed positive effects of structural social capital on relational and cognitive social capital, and the mediation role of relational social capital was demonstrated. We also tested the effects of differences in sociodemographic characteristics. Our findings verify the explanatory power of social capital regarding residents’ complex relationships in neighborhood renewal in China. Implications for theory and policy are discussed. This study helps to improve our understanding of residents’ social systems in neighborhood renewal and provides theoretical support for formulating neighborhood renewal policies in China and abroad.

## 1. Introduction

The concept of neighborhood renewal has attracted the attention of urbanists in recent years. This is not surprising, given the increasingly important role of neighborhood renewal in rejuvenating dilapidated districts (Troy et al., 2015). In China, neighborhood regeneration is becoming an important strategy for urban development. Over 42 million households live in China’s old neighborhoods; in 2020 alone, 39,000 old neighborhood renewal projects involved seven million residents (Liu et al., 2022). Regarding such projects, the Chinese government has promoted the idea that “cities are built by the people and for the people,” as it aims to improve the well-being of neighborhood residents. The importance of residents in this process has been recognized by not only the central Chinese government but also other regional and national governments. In England, for example, having pursued various forms of neighborhood renewal since 1997, the government came to recognize the important role of resident characteristics and behaviors in renewal projects (Lupton et al., 2013). Thus, residents are being viewed as the protagonists of neighborhood renewal in cities around the world.

In China, however, residents have specific characteristics that are mainly reflected in the relationships between them. First, relationships between residents are historical. Under the pre-1980 centrally planned economy, many neighborhoods (now known as “old neighborhoods”) were built and provided by danwei, which is defined as an organization (e.g., a department, section, or battalion) that is considered a subunit of a larger organization (Andrew, 1988; Xie and Costa, 1993; Bo, 2014). Many residents in a neighborhood may be colleagues or even subordinates. In community life, colleague relationships or superior–subordinate relationships can gradually evolve into either friendships or hostile relationships. At the same time, the movement of people can bring new residents to neighborhoods, which creates new types of relationships. These “historical” factors in older neighborhoods can therefore lead to complex resident relationships.

Second, resident relationships influence decision-making about renewal. This can be considered the “political” dimension of resident relationships. Policies in many Chinese cities stipulate that before a community renewal program can be executed, a certain percentage of the residents must agree to it (The People’s Government of Shantou Municipality, 2014; The People’s Government of Guangzhou Municipality, 2016). However, residents’ perceptions of renewal projects are easily influenced by those around them (Dodoiu, 2015; Xu et al., 2017). In other words, residents may make decisions based not only on their own interests, but also on their relationships. In the context of neighborhood renewal, such relationship-influenced decision-making can determine whether a renewal project is implemented.

Third, the different social structures in a neighborhood can lead to heterogeneous relationship structures. In China, young people face increasing pressure to purchase houses, and older neighborhoods are gaining popularity because of their lower prices and generally good locations. As a result, older neighborhoods have increasingly heterogeneous social structures (e.g., some have more young people while others have more older people). This phenomenon makes it impossible to regard the social relationships in neighborhoods with different social structures as belonging to the same type, making it necessary to consider whether they need to be analyzed separately.

The abovementioned historical, political, and heterogeneous characteristics of resident relationships highlight the need for further research on the social relations of residents in China’s older neighborhoods. Social capital theory was adopted as the foundation of this study due to the complexity of relationships. As the term implies, social capital is the resources embedded in relationships that can be used to benefit oneself (Zhai and Ng, 2013; Appau et al., 2022), and it has been widely used to analyze resident relationships. However, the “social capital” of Chinese residents examined in this study differs from that of previous studies. First, we focused only on the effect of social capital on neighborhood renewal. Previous studies have suggested that there should be an interplay between neighborhood renewal and community social systems. Preexisting social relationships in the community can facilitate or hinder the implementation of renewal projects. Conversely, renewal projects can disrupt or restructure social relationships in a community. This study focused only on the former. However, because of the complexity of relationships among residents in Chinese old neighborhoods, we were more interested in exploring how the multiple types of existing social relationships—or multidimensional social capital—among residents influenced the implementation of neighborhood renewal. Second, this study focused only on the social capital of residents. Previous research, meanwhile, has mainly investigated the relationships between different parties, such as governments, cooperates, and resident groups. Such research tends to regard residents as a homogenous group. As mentioned above, however, because of the different interests and complex relationships among residents, the resident group should be viewed as heterogeneous. In other words, the conflicts and complex relationships among residents were the focus of this study. To our knowledge, few studies have investigated these conflicts and complex relationships among residents based on social capital.

This study aimed to better understand resident relationships in neighborhood renewal in China based on social capital. We conducted a survey to collect data from 590 residents across China who were experiencing or had experienced neighborhood renewal. Social capital theory provided the basic theoretical framework for analyzing resident relationships. Specifically, we addressed the following questions:

1. How can we distinguish and describe the different types of social relations among residents from the perspective of social capital?
2. How do the different types of social relations (or different types of social capital) among residents interact with each other?
3. How do sociodemographic characteristics (e.g., age, gender, length of residence, income) influence residents’ social capital?

The remainder of this article is structured as follows. Following the literature review, the theoretical framework is established, and the hypotheses are developed. Section 3 describes the data collection and analysis procedures. Sections 4 and 5 report and discuss the results, respectively. Section 6 concludes the article.

## 2. Literature review and hypothesis development

There has been previous research on the impacts of covariates on individual social capital in a variety of contexts. There is a lack, however, of an integrated model that can synthesize the relationships between residents’ multidimensional social capital. In what follows, we first review research on social capital theory and multidimensional social capital in neighborhood renewal. Then, we review research on the effects of covariates on individual social capital, which we use to justify the theoretical validity of using structural equation modeling (SEM) and multiple indicators multiple causes (MIMIC) modeling.

### 2.1. Social capital theory

Previous research has found that individuals embedded in social networks can gain support through mutually cooperative behavior with others in the network, which can be considered one kind of resource (Phua et al., 2017). Social capital refers to tangible or intangible resources that people can gain from their social connections (Spottswood and Wohn, 2020). Since Hanifan (1916) early work on social capital, a large and growing body of research has focused on the concept, drivers, and effects of social capital. Collective action and cooperation are enabled by social capital, resulting in positive outcomes (Knack and Keefer, 1997). Positive results generated include the following: (i) the opportunity to access more information and increase the quality of the acquired knowledge (Randolph et al., 2020), (ii) reductions in transaction costs (Zhao J. et al., 2021), (iii) social support and social incentives for individual development (Lukasiewicz et al., 2019), and (iv) a potentially more efficient allocation of resources (Zhao Y. et al., 2021). Based on social capital literature, we define the social capital of residents in neighborhood renewal as the sum of actual and potential resources, transmitted through social relationship networks, available to residents during neighborhood renewal (Lukasiewicz et al., 2019).

Researchers regard neighborhoods as diffuse networks of personal relationships; thus, social capital is viewed as the value of these relationships in the lives of individuals (De Zuniga, 2016). Social capital can also be defined as localized social capital according to Giudici et al. (2018). Localized social capital, such as residents’ social capital, represents the resources individuals can access *via* membership in a group or community (Cramm et al., 2013). Social capital, in this sense, can reflect the strength of ties between residents and their neighbors and friends, as well as the level of trust and reciprocity norms among residents (Sadri et al., 2018). In the context of neighborhood renewal, social capital can be considered a driving force for the successful implementation of renewal plans (Shen et al., 2021). Previous research have shown that social capital can raise levels of local trust, social support, and norms (McNeill et al., 2006; Poortinga, 2012; Collins et al., 2014). Cox (2000) suggested that the higher the level of social capital in a neighborhood, the better its ability to manage difficulties. Izadi et al. (2020) similarly proposed that residents with higher social capital will have a stronger sense of belonging and will be more willing to participate in neighborhood renewal, which will make the project more effective. Other studies, meanwhile, have shown that high social capital can complicate decision-making processes, which will influence the outcomes of neighborhood renewal (Halpern, 2005). Given the prevalence of residents’ social capital and its profound impact on the performance of neighborhood renewal projects, it is of practical and theoretical significance to study the mechanisms of residents’ social capital’s role in neighborhood renewal.

Putnam (1995) proposed that social capital is multifaceted, and Meng et al. (2018) likewise characterized it as a multidimensional concept. There are currently two ways of classifying social capital—namely, from either structural or functional perspectives. In the structuralist view, social capital is normally regarded as outcomes generated from an individual’s social network (Bourdieu, 2007). Previous research adopting this perspective has classified social capital as bonding (social ties within homogeneous groups), bridging (social ties within heterogeneous groups), and linking social capital (links between different power classes) (Putnam, 1995; Szreter and Woolcock, 2004). Under functionalism, meanwhile, social capital can be seen as one kind of value facilitating certain actions among group members (Coleman, 1988). Nahapiet and Ghoshal (1998) divided social capital into three aspects: structural social capital (overall relationships or connections among social actors), relational social capital (relations embedded in social actors’ bonds, including factors like trust and trustworthiness), and cognitive social capital (common goals or shared interests among social actors). In the context of neighborhood renewal, the relationship between residents is not only about the communication of information or the distribution of power between residents. In other words, the social capital of residents is not equal to the social structure of the resident group. On the contrary, it was trust, reciprocity, and social norms among residents that promote community cooperation (Du et al., 2020; Zugravu-Soilita et al., 2021; Shin, 2022). Thus, analyzing residents’ social capital from a functional perspective can provide a more comprehensive understanding of the multiple attributes of residents’ social relationships than from a structural perspective. This study, therefore, chose to classify social capital from a functionalist perspective and analyze the inherent mechanisms of structural, relational, and cognitive social capital.

### 2.2. Structural social capital in neighborhood renewal

Structural social capital (SSC) can be defined as the pattern of actors’ connections with each other in a social system (Nahapiet and Ghoshal, 1998). SSC encompasses several components, such as the hierarchies in social networks and the formal and informal connections within organizations (Muniady et al., 2015). SSC develops social structures and facilitates information sharing and cooperation through actors and relationships under defined rules and procedures (Alan and Köker, 2021). Information exchange is the core component of SSC (Lawson et al., 2008; Yim and Leem, 2013; Li et al., 2022). García-Villaverde et al. (2017) suggested that SSC emphasizes the control of information in the social system structure.

In the context of neighborhood renewal, SSC refers to residents’ neighborhood interactions, discussion networks, and social participation (Yuan, 2016). Increasing community cohesion and participation in revitalization programs need the creation of links and networks within communities (Card and Mudd, 2006). Regarding governments and cooperation, previous studies have mainly focused on information sharing and collaboration within organizations (Atabakhsh et al., 2004). In the domain of neighborhood renewal, interactions among the government, developers, and residents have received the most attention (Jung et al., 2015; Chu et al., 2020). There is, however, a lack of research specifically investigating relationships within the resident group. Previous studies have indicated that residents typically build dense networks through regular contact with neighbors and friends, which plays an important role in social network formation (Lelieveldt, 2004; Moreira de Souza, 2019). In other words, heterogenous groups may also arise as a result of differing opinions among residents. Communication and interaction (i.e., SSC) among residents are also importance of cooperation in neighborhood renewal.

Measuring SSC is not widely agreed upon. Indeed, Lu and Peng (2019) suggested that there is no common understanding regarding the measurement of SSC, which makes it hard to make comparisons between the results of different studies. Commonly used measures for SSC include social network analysis, questionnaires, and semi-structured interviews (García-Villaverde et al., 2018a; Wong et al., 2018; Shin, 2022). In older neighborhoods, however, the researcher as a stranger cannot easily obtain trust, which can make it difficult for the researcher to obtain data from residents. Therefore, constructing a detailed social network of residents is complicated and poses certain difficulties. Shin (2022) similarly noted that social capital is private in nature, and obtaining sufficient data to develop a social network will be difficult if there is no social capital between researchers and respondents. Shin (2022) therefore developed a social network model that includes only 16 residents, which is obviously much lower than the actual number of residents in a neighborhood or even a building (Shin, 2022). In light of such considerations, we chose to use a questionnaire to measure SSC of residents through multiple observed variables. With reference to Vera-Toscano et al. (2013), Zhang et al. (2015), Qian et al. (2018), and Wong et al. (2018), we measured SSC in terms of the diversity of pattern, frequency, and broad connection.

### 2.3. Cognitive social capital in neighborhood renewal

Cognitive social capital (CSC) is one type of resource embedded in a social system, generated from a common system of representations, explanations, and meanings (Nahapiet and Ghoshal, 1998). The level of agreement among people in a social network is known as CSC (Goode, 2021). It is derived from a shared language and code that promotes the development of common understanding in interaction (Kim et al., 2020). CSC originates heavily from the relational norms of behavior in a shared mental context (Goode, 2021). In other words, people who share similar values and viewpoints are more likely to be attracted to one another (Jonsson, 2015). García-Villaverde et al. (2018a) suggested that CSC can help to reduce transaction costs and solve coordination problems. Westerlund and Svahn (2008) highlighted the positively association between CSC and relationship value. Others, meanwhile, have considered negative aspects of CSC, such as the tendency toward exclusion and keeping outsiders at bay in the social system (Jonsson, 2015). According to Petruzzelli (2011), CSC and project performance have an inverted U-shaped relationship. CSC plays an important role in social systems, whether it has positive or negative effects (Kwon and Adler, 2014).

There are different aspects of CSC, and each can have its own effects on the development of CSC (Wong et al., 2019). Rodrigo-Alarcón et al. (2020) proposed that CSC includes elements that contribute to information exchange among members, such as interests, and norms. Hung and Lau (2019) suggested that sense of community and trust are two major components of CSC. Steinmo and Rasmussen (2018) classified CSC into shared goals and culture—a classification that has been adopted by subsequent researchers. Shared goals can be regarded as common views and understandings regarding how to achieve tasks and goals (García-Villaverde et al., 2018b). Shared culture, meanwhile, can be regarded as the common rules and norms that determine appropriate behavior (Steinmo and Rasmussen, 2018).

In the context of neighborhood renewal, shared goals are the most important aspect of CSC. This is because diverse interests among residents can hinder the generation of consensus on neighborhood renewal projects (Dargan, 2009). In the course of community life, there are few public affairs that require the participation of most or all residents, which means that residents rarely need to work toward common goals. Therefore, different from corporate employees, residents are unable to quickly balance interests within their system when faced with a task (such as neighborhood renewal) (Liu et al., 2021). This makes it easier for conflicts of interest to arise between residents. It is clear that there is no way to completely eliminate the diversity of interests among residents. Existing studies have mostly have focused on how to bridge the gap between different interest groups (Deboulet and Abram, 2017; Liu et al., 2020; Kabalo and Etkin, 2022). On the one hand, this approach aims to facilitate mutual respect and balance among residents with different interests. On the other hand, it aims to improve project performance by fostering common goals among residents. Therefore, in the context of neighborhood renewal, residents’ CSC mainly refers to their common views and understandings regarding neighborhood renewal.

Social capital has multiple dimensions that are interconnected (García-Villaverde et al., 2018a). Qian et al. (2018) found a curvilinear, rather than linear, relationship between SSC and project performance; the reason could be that SSC’s interaction with other dimensions of social capital had previously been ignored. Other studies have reached similar conclusions. According to Muniady et al. (2015), SSC and CSC are significantly related. Lu and Peng (2019) proposed that the structural dimension is closely related to the cognitive dimension. A community’s CSC can be improved by SSC, according to Lu and Lou (2022). In the context of neighborhood renewal, high SSC can help residents communicate with their neighbors, which may facilitate common understanding regarding renewal projects. Hence, the following hypothesis is proposed:

*H1*: Structural social capital is positively associated with the cognitive social capital of residents in neighborhood renewal.

### 2.4. Relational social capital in neighborhood renewal

The strength of relationships between members of a social system is referred to as relational social capital (RSC) (Nahapiet and Ghoshal, 1998). It can measure an individual’s ability to obtain valuable resources through social relationships and group membership (Yee, 2015). RSC, then, is the effect of cultivating strong relationships that lead to positive outcomes (Grawe et al., 2014). Previous research has described RSC as a kind of “glue” in social systems (Grawe et al., 2014). Pinho (2013) proposed that the relational dimension enables cooperation by fulfilling a number of social needs for people, such as sociability and prestige.

Of the three dimensions of social capital, RSC is particularly attractive because it describes the instability of human relationships (Sadik and Wee, 2018). Unlike the structural dimension, the relational dimension does not arise from the diversity or frequency of communication, and unlike the cognitive dimension, it does not arise from perceptions of specific things. Rather, it approximates a description of the way in which individuals make friends. RSC is partly influenced by historical and cultural contexts (Elfenbein and Zenger, 2014). Son and Feng (2019), for example, found that in China, a dense network structure is crucial for trust generation (which is the most critical component of RSC); in the US, however, psychological closeness is more important. This explains why it is meaningful to examine social capital in the Chinese context. In China, the concept of *guanxi* can be used to describe RSC; specifically, it refers to getting acquainted to gain benefits in personal relationships (Zheng et al., 2014). DiTomaso and Bian (2018) proposed that in China, *guanxi* is generated from family, kin, and friendships; it is seen as familial, or pseudo-familial, and involves clearer expectations about human exchange. In the context of neighborhood renewal, Zhai and Ng (2013) defined *guanxi* as the connection between residents, which involves the realization of self-interest. Barnes et al. (2011) proposed that *guanxi* includes *ganqing* (affection between people), *xinren* (trust), and *renqing* (favor). Likewise, this study measured RSC in those three dimensions.

As mentioned above, there are relationships between the different aspects of social capital. First, SSC has been shown to have an effect on RSC. Paxton and Moody (2003) suggested that the social networks people are in may influence how people feel about each other. Zhang et al. (2018) suggested that interactions and ties between people will affect the characteristics and quality of relationships. Lu and Peng (2019) noted that SSC can enrich social trust among people. In the context of neighborhood renewal, it can be difficult to develop trust and affection between residents without communication. Further, according to Lin (2018), interaction is the basis for generating affection. Residents who communicate frequently are more likely to develop friendships. Hence, we propose the following:

*H2*: Structural social capital is positively associated with the relational social capital of residents in neighborhood renewal.

Second, according to previous studies, CSC and RSC can be related. According to Qian et al. (2018), RSC can reduce conflict. In other words, relational dimensions may help to achieve shared goals. Men et al. (2020) likewise suggested that RSC can motivate group members to work together toward shared goals and promote cooperative norms. High levels of trust can help avoid opportunistic behavior and facilitate collaboration (Steinmo and Rasmussen, 2018). Previous studies of neighborhood renewal have emphasized the “neighborhood effect” (Dodoiu, 2015; Xu et al., 2017). This refers to the phenomenon in which the existence of a preferred collaborative approach in the community may increase residents’ willingness to adopt collaborative behavior (Mathers et al., 2008). We therefore propose the following:

*H3*: Relational social capital is positively associated with the cognitive social capital of residents in neighborhood renewal.

Regarding the relationship between the three dimensions, Liao and Welsch (2005) proposed that SSC occupies the most fundamental position among them. Further, Jonsson (2015) suggested that without the SSC, RSC is less likely to develop, and the generation of the CSC will be hampered. Similarly, Zheng (2010) argued that SSC is closely related to RSC, which in turn promotes a shared vision. According to Carey et al. (2011), RSC mediates the relationship between SSC and CSC. Therefore, we propose the following hypothesis:

*H4*: Relational social capital mediates the relationship between the structural and cognitive social capital of residents in neighborhood renewal.

### 2.5. Age, gender, length of residence, and income differences in residents’ social capital in neighborhood renewal

Sociodemographic characteristics (e.g., age, gender, length of residence, income) are also considered potentially important personal characteristics with regard to residents’ social capital. Mao et al. (2022) noted the effect of different types of neighborhood environments on social capital. Meanwhile, questions have been raised about whether social capital varies across genders (Leeves and Herbert, 2014). Addis and Joxhe (2017) proposed that the life cycle of social capital accumulation differs for male and female residents. Women and men share the same importance of neighborhood social capital, according to Eriksson and Emmelin (2013). Regarding age, previous studies have identified differences in social capital according to age (Neves, 2015). McDonald and Mair (2010) found that daily interaction was negatively correlated with age. Nyqvist et al. (2016) confirmed the moderating role of age in social contact. Regarding length of residence, Siordia and Saenz (2013) suggested that residents with a long length of residence will have more positive neighborhood perceptions. Yamamura (2011) found that length of residence positively influenced investment in social capital. Finally, with regard to income, researchers are concerned with whether income moderates the effect of social capital on other elements (such as satisfaction) (Zhang, 2022). Lu et al. (2021) found that income moderates the relationship between SSC and CSC. Hermann and Kopasz (2011) found that in most countries and regions, there were significant positive associations between income and social capital.

In summary, differences in sociodemographic characteristics such as age, gender, length of residence, and income can be said to shape social capital (Kwon and Adler, 2014). Nevertheless, we find that there remains inconsistent empirical evidence supporting the effects of these sociodemographic characteristics. Therefore, we used MIMIC modeling to examine the latent theoretical constructs, controlling for the impact of the abovementioned sociodemographic characteristics. To conclude, we propose the following hypothesis:

*H5*: Gender has the effects on social capital of residents (relational, structural, and cognitive) in neighborhood renewal.

*H6*: Age has the effects on social capital of residents (relational, structural, and cognitive) in neighborhood renewal.

*H7*: Length of residence has the effects on social capital of residents (relational, structural, and cognitive) in neighborhood renewal.

*H8*: Income has the effects on social capital of residents (relational, structural, and cognitive) in neighborhood renewal.

## 3. Methods

### 3.1. Participants and procedure

The investigation was conducted between October 2021 and January 2022. The survey was conducted in three cities in China: Chongqing, Nanjing and Xuzhou. All three cities have published lists of pilot projects for neighborhood renewal, and this study distributed questionnaires to residents of the pilot projects based on these lists. Another reason for choosing these three cities for the study is to cover as many city types as possible. Chongqing is located in western China, while Nanjing and Xuzhou are located in eastern China. In addition, Chongqing and Nanjing are among the first-tier cities in China, and Xuzhou is among the second-tier cities in China. The number of interviewed residents in the three cities was designed to be as equal as possible at the beginning of this study. However, due to research costs and time constraints, the final sample sizes obtained in Nanjing and Xuzhou were slightly smaller than those in Chongqing. This is also because some neighborhoods in Nanjing and Xuzhou are difficult for the authors to enter the neighborhoods to distribute the questionnaires because of the anti-epidemic policy. Finally, this study obtained a total of 328 questionnaires in 24 neighborhoods in Chongqing, 203 questionnaires in 19 neighborhoods in Nanjing, and 192 questionnaires in 15 neighborhoods in Xuzhou.

For the survey, the target population was residents in neighborhoods that were either currently undergoing renewal, about to begin renewal, or had completed renewal. Before the survey, participants were briefed about the questionnaire and ensured that their personal information would remain confidential. Several demographic questions and a social capital scale were asked of residents. A total of 723 residents participated in the survey, of which 590 completed the questionnaire and provided valid questionnaires (valid response rate: 81.6%). Previous studies have indicated that certain demographic factors (e.g., gender, and age) can significantly affect residents’ social capital. Thus, gender, age, length of residence, and income were measured in this study.

The gender distribution of respondents was 55.1% (*n* = 325) male; the rest were female. Age ranged from 19 to 78 years. Regarding length of residence, 64.6% (*n* = 381) had lived in their neighborhood for more than 10 years. The majority (83.1%, *n* = 490) had a monthly household income of 2,500–10,000 CNY while only 17.0% (*n* = 100) had a monthly household income lower than 2,500 CNY or higher than 10,000 CNY.

### 3.2. Measures

According to Ehsan and De Silva (2015), social capital can be measured by asking respondents about their perceptions of relationships. A multiple-item questionnaire was used that included questions about SSC, RSC, and CSC, as well as demographics. Based on the Likert scale of 1 to 7 (strongly disagree, strongly agree), statements were rated. All scales had been validated in previous studies.

#### 3.2.1. Structural social capital

A three items scale developed by Zhang et al. (2015) and Vera-Toscano et al. (2013) was used to measure SSC. Sample items include “We often invite our neighbors to attend informal social events (e.g., walk, chat, or public square dance)” (diversity of pattern), “Our residents frequently interact with each other” (Frequency), and “I know most people in the neighborhood” (broad connection).

#### 3.2.2. Cognitive social capital

Respondents rated CSC on a four-item scale developed by Zhang et al. (2015) and Chen and Hung (2014). Sample items include “Our residents have the same views on neighborhood renewal,” “Our residents have the same interest pursuits regarding neighborhood renewal,” “Our residents care about not only their own benefit but also others’ interests,” and “Our residents have common understandings about concepts related to neighborhood renewal.”

#### 3.2.3. Relational social capital

**Figure 1:**
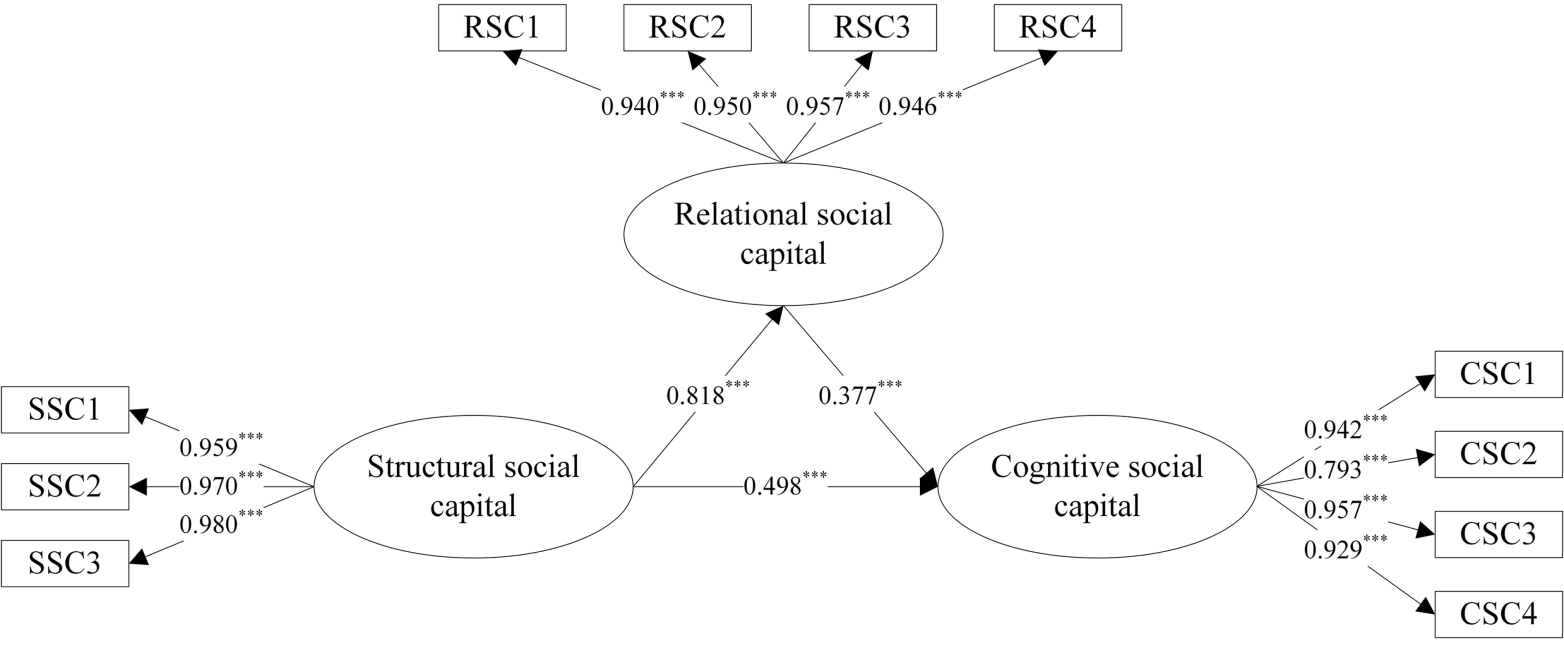
Direct effect results. ***p<0.001.

Residents rated RSC on a four-item scale from Lee (2015) and Vera-Toscano et al. (2013). Sample items include “I like to spend time with my neighbors” (*ganqing*), “Residents in my neighborhood look after others’ needs and interests” (*ganqing*), “Most residents trust each other” (*xinren*), and “If I help my neighbors, my neighbors will help me when I need it” (*renqing*) (Figure 1).

### 3.3. Analytical procedures

Statistical analysis, preliminary descriptive analysis, and hypothesis testing were performed using SPSS (version 20.0) and Mplus (version 8.3).

Data were analyzed using SEM and MIMIC modeling, which are becoming increasingly popular in research on residents’ psychology (e.g., Cao et al., 2007; Wang and Chai, 2009; Cervero and Murakami, 2010). SEM can test propositions for one-way or correlated relationships between variables (Anderson and Gerbing, 1988). MIMIC can observe the restriction of the covariance matrix for the observed indicators (Jöreskog and Goldberger, 1975). We followed the SEM and MIMIC procedures suggested by Proitsi et al. (2011). Our first step was to confirm the factor structure of the three constructs through confirmatory factor analysis (CFA). Second, the structural relationships between the three constructs were tested. Third, MIMIC was used to test the covariates’ effects on the factor structure of residents’ multidimensional social capital (Table 1).

**Table 1:** Characteristics of the respondents.

Variables	*n* = 590	Percentage %
**Gender**		
Male	265	44.9
Female	325	55.1
**Age**		
≤30	50	8.5
31–50	216	36.6
≥50	324	54.9
**Length of residence**		
≤3 years	37	6.3
4–9 years	172	29.2
≥10 years	381	64.6
**Monthly household income**		
≤2,500 CNY	40	6.8
2,500–5,000 CNY	204	34.6
5,000–10,000 CNY	286	48.5
≥10,000 CNY	60	10.2

## 4. Results

### 4.1. Reliability and validity testing

To test the fit of the measurement model, CFA was conducted. A good fit was found between the hypothesized three-factor model and the data in Table 2 [*χ*^2^ (41) = 99.400, *p* < 0.001; RMSEA = 0.049; CFI = 0.994; TLI = 0.992; SRMR = 0.021]. Using a common factor analysis, all parceled items were loaded onto a single factor representing the “methods factor” in order to eliminate common bias concerns.

**Table 2:** Results of confirmatory factor analysis.

Model	$\chi^{2}\left(df\right)$	$\Delta \chi^{2}$	CFI	TLI	SRMR	RMSEA
Three-factor model	99.400^***^(41)	-	0.994	0.992	0.021	0.049
One-factor model	2556.492^*^(44)	2457.092	0.747	0.683	0.069	0.311

*p < 0.05, **p < 0.01, ***p < 0.001.

A measurement model’s reliability, convergent validity, and discriminant validity were assessed. Table 3 shows that the constructs (i.e., SSC, RSC, and CSC) all demonstrated good reliability, with Cronbach’s alpha values all higher than the threshold of 0.8 (Nunnally, 1978). Three indices were used to assess convergence validity: factor loading, average variance extracted (AVE), and construct reliability (CR). The convergent validity results are also shown in Table 3. Factor loadings and the AVEs were all above 0.5, and the CRs were all above 0.7 (Fornell and Larcker, 1981), which prove that convergent validity was acceptable.

**Table 3:** Reliability and validity test results for the variables.

Construct	Indicator	Item value: scale	Factor loading	Cronbach’s α	CR	AVE
Structural social capital	SSC1	Strongly disagree → Strongly agree: 1 → 7	0.959	0.979	0.979	0.941
	SSC2	Strongly disagree → Strongly agree: 1 → 7	0.970			
	SSC3	Strongly disagree → Strongly agree: 1 → 7	0.980			
Relational social capital	RSC1	Strongly disagree → Strongly agree: 1 → 7	0.940	0.972	0.973	0.900
	RSC2	Strongly disagree → Strongly agree: 1 → 7	0.950			
	RSC3	Strongly disagree → Strongly agree: 1 → 7	0.957			
	RSC4	Strongly disagree → Strongly agree: 1 → 7	0.946			
Cognitive social capital	CSC1	Strongly disagree → Strongly agree: 1 → 7	0.942	0.944	0.949	0.824
	CSC2	Strongly disagree → Strongly agree: 1 → 7	0.793			
	CSC3	Strongly disagree → Strongly agree: 1 → 7	0.957			
	CSC4	Strongly disagree → Strongly agree: 1 → 7	0.929			

A correlation between variables and the square root of AVE can be used to verify discriminant validity. The square root of a variable’s AVE should be all above the correlation coefficients involving that variable (Fornell and Larcker, 1981). There was an acceptable level of discriminant validity as shown in Table 4.

**Table 4:** Results for validity and descriptive statistics.

Variables	Mean	1	2	3
1. Structural social capital	4.316	**0.970**		
2. Relational social capital	4.177	0.773	**0.907**	
3. Cognitive social capital	4.632	0.799	0.746	**0.949**

The diagonal numbers are the square root of AVE; the remaining numbers are the Pearson’s correlation coefficients.

### 4.2. Descriptive and correlational statistics

The descriptive statistics of the studied variables are presented in Table 4. The Pearson’s correlation coefficients indicated that SSC correlated significantly with RSC (*r* = 0.773, *p* < 0.01) and CSC (*r* = 0.799, *p* < 0.01). RSC correlated significantly with CSC (*r* = 0.746, *p* < 0.01).

### 4.3. Hypothesis testing

Table 5 shows the standardized estimates. All items were significant (*p* < 0.001). The variations in CSC and RSC are explained by 69.8 and 67.0%, respectively. Table 5 also shows that SSC had a positive impact on CSC (*β* = 0.498, *p* < 0.001) and RSC (*β* = 0.818, *p* < 0.001), thus supporting H1 and H2. RSC had a positive impact on CSC (*β* = 0.377, *p* < 0.001), supporting H3. Furthermore, the indirect effects of SSC on CSC through RSC were significant (*β* = 0.315; SE = 0.045; 95% CI = [0.225, 0.401]), thus supporting H4. We can conclude, therefore, that RSC mediates the effects of SSC on CSC.

**Table 5:** Effects of covariates on latent theoretical constructs in the MIMIC model.

Dependent variable	Predictors and covariates	*β*	S.E.	*p* > |*z*|
Structural social capital	Gender	0.002	0.040	0.970
	Age	−0.020	0.060	0.739
	Length of residence	0.203	0.056	***
	Income	0.108	0.038	**
Relational social capital	Gender	0.026	0.027	0.339
	Age	−0.110	0.034	**
	Length of residence	−0.087	0.036	*
	Income	−0.034	0.029	0.241
Cognitive social capital	Gender	0.001	0.023	0.977
	Age	−0.004	0.032	0.906
	Length of residence	0.003	0.031	0.933
	Income	0.050	0.027	0.065

*p < 0.05, **p < 0.01, ***p < 0.001; SE, standard error.

### 4.4. MIMIC model

**Figure 2:**
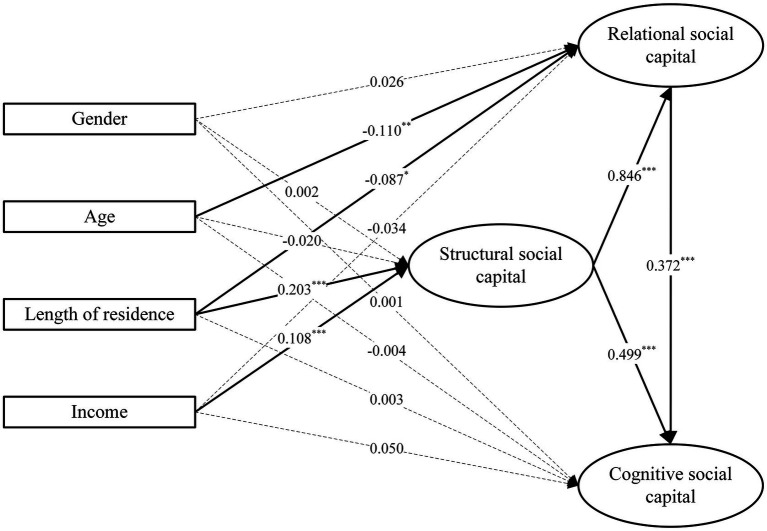
MIMIC model with the effects of the covariates on the latent theoretical constructs. *p < 0.05, **p < 0.01, ***p < 0.001; dashed lines = non-significant.

MIMIC incorporated gender, age, length of residence, and income as covariates. The MIMIC model had good model fit [*χ*^2^(61) = 132.963, *p* < 0.001; RMSEA = 0.045; CFI = 0.993; TLI = 0.988; SRMR = 0.018]. Based on the MIMIC model, Table 5 shows how the covariates affect the latent constructs. Figure 2 visually depicts the MIMIC model. Gender had no statistically significant effect on SSC, RSC, or CSC in the MIMIC model. Age had a negative but small impact on RSC (*β* = −0.110, *p* < 0.01). Length of residence had a significant impact on both SSC (*β* = 0.203, *p* < 0.001) and RSC (*β* = −0.087, *p* < 0.05). Income had a positively effect on SSC (*β* = 0.108, *p* < 0.01). Overall, the MIMIC model explained 4.6% of the variation in SSC, 69.9% of that in CSC, and 69.6% of that in RSC.

## 5. Discussion

There has been an increasing interest from researchers and practitioners around the world in the social capital of residents involved in neighborhood renewal. The current study investigated a theoretical framework for the multidimensional social capital (including SSC, CSC, and RSC) of Chinese residents facing neighborhood renewal. Specifically, we aimed to determine (1) how can we distinguish and describe the different types of social relations among residents from the perspective of social capital, (2) how do the different types of social relations (or different types of social capital) among residents interact with each other, and (3) how do sociodemographic characteristics (e.g., age, gender, length of residence, income) influence residents’ social capital. To that end, using social capital as the basic theory, we designed a questionnaire and collected data from 590 residents across China who were experiencing or had experienced neighborhood renewal.

### 5.1. Theoretical implications

This study empirically demonstrated the theoretical framework of multidimensional social capital. First, we show that SSC is positively associated with both RSC and CSC. In line with Castro and Roldán (2013), we confirmed that maintaining close social relationships may improve the development of mutual trust and respect. As Nonino (2013) noted, dense relationships create opportunities to perceive similarities with other people and foster closer relationships. Our results also supported García-Villaverde et al. (2018b), who found that SSC is important of CSC. Second, we demonstrated that RSC is positively related to CSC. Residents’ personal opinions can be influenced by friends, family, and neighbors in their neighborhood. In other words, residents may learn from and copy the behaviors and decisions of the people around them (Dodoiu, 2015; Xu et al., 2017).

Contrary to previous studies, SSC had a greater effect on RSC than CSC in this study (Zhang et al., 2015; Chang and Hsu, 2016; García-Villaverde et al., 2018b). This could have to do with the special characteristics of resident groups. Most previous research mainly focused on formal organizations such as tourism organizations (Chowdhury et al., 2019) and business–customer relations (Zhang et al., 2015). This study highlighted the differences between resident groups and formal organizations from the perspective of conflict. Conflict has been classified into two types: task and relationship conflicts (Ma et al., 2008). Task conflict refers to different viewpoints regarding a team assignment; relationship conflict refers to interpersonal incompatibility (Wu et al., 2017). De Wit et al. (2012) proposed that, in a company, task conflict may improve innovativeness and group decision-making while relationship conflict may harm group performance. Horak et al. (2020) suggested that informal networking in a company may lead to collusion, cliques, and corrupt conduct. Therefore, information exchange and connections within formal organizations mainly concern the achievement of shared goals, not the establishment of close relationships between employees. This is different from resident groups, where the establishment of information exchange relationships among residents precedes the emergence of the task of neighborhood renewal. In the course of community life, residents cultivate and consolidate the people with whom they exchange information. In other words, frequent communication turns neighbors into friends. For residents, therefore, connections with neighbors are more about friendship than the completion of renewal tasks.

The effect of SSC on CSC was stronger than that of RSC. In the study context (i.e., the effect of established social systems in neighborhoods on neighborhood renewal), this result is surprising, because it suggests that while friendship and trust among residents might have some effect on shared goals, they are not that important. One possible reason is that the role of RSC is weakened by conflicts among residents arising from diverse interests. Neighbors formerly on good terms might turn against each other because of different interests regarding neighborhood renewal. Meanwhile, residents who were previously strangers might have the opportunity to exchange information in the process of neighborhood renewal. Even if this frequent level of communication is not enough to develop friendships and trust among residents, it can help form shared goals. It does not mean, however, that RSC is not important for CSC formation. RSC was found to mediate the effects of SSC on CSC. Accordingly, through RSC, SSC influences CSC. Therefore, the relationship between RSC and CSC was significant in this study, and its role should not be underestimated.

Regarding sociodemographic characteristics, gender had a nonsignificant effect on all dimensions of social capital. This diverges from previous studies that identified gender differences in social capital (Son and Lin, 2008; Moore and Carpiano, 2020). One possible reason is that previous studies focused more on gender differences in political or corporate systems (Matsunaga, 2015). Compared with political and corporate settings, neighborhood residents are less likely to consciously invest in and earn social capital in their community life. As a result, gender differences are less pronounced in the context of neighborhood renewal.

Consistent with Zhou et al. (2022) and Menardo et al. (2022), we found significant age differences with regard to RSC. We should note, however, that age differences might not affect SSC and CSC. In other words, age differences might affect whether residents trust each other but not the exchange of information and the formation of shared goals during neighborhood renewal.

This study also found that differences in length of residence had effects on residents’ SSC and RSC. This is in line with previous research (Yamamura, 2011; Siordia and Saenz, 2013). The longer residents live in a neighborhood, the more likely they are to communicate with neighbors and develop trust. This also explains why differences in length of residence did not influence CSC. Neighborhood renewal appears as a short-term project or task for resident groups. Therefore, length of residence should not have an effect on residents’ interest in neighborhood renewal.

Unlike length of residence, income differences only affected SSC. Consistent with Hermann and Kopasz (2011), residents with higher incomes were more likely to associate with other residents. However, we found that high-income residents appeared to only associate with other residents, and they rarely established substantial friendships or otherwise influenced the formation of common goals.

By revealing the connections between SSC, RSC, and CSC and the effects of sociodemographic characteristics, this study clarified the interrelationships among the multidimensional social capital of residents, thus providing a comprehensive picture of a theoretical model of residents’ social capital in neighborhood renewal.

### 5.2. Policy implications

There are also important policy implications for neighborhood renewal resulting from our findings. First, social relations among residents can be considered a policy tool for implementing neighborhood renewal. We confirmed the effect of RSC on CSC and the mediating role of RSC. The more friends residents have in their neighborhoods, the more likely they are to reach consensus with their surrounding neighbors regarding neighborhood renewal. This implies that the level of RSC among residents can be used as an indicator to assess whether an old neighborhood is feasible for renewal. It is also necessary to create certain management positions for residents with high social capital. Considering differences in length of residence and income, local governments and firms can seek cooperation from high-income residents who have lived in the neighborhood for a long time.

Moreover, if the general level of neighborhood residents’ social capital is not high, the preparation phase of neighborhood renewal can be extended appropriately. We confirmed that in addition to RSC, SSC (e.g., information exchange and connections) can also facilitate consensus formation. SSC can also facilitate the formation of trust among residents. Therefore, local governments can plan to promote interactions among residents (e.g., through cultural and entertainment activities) before implementing neighborhood renewal projects. Local governments can wait until the relationships among residents are on good terms before implementing renewal projects. We suggest, therefore, that there is a need to reconfigure the whole life cycle process of old neighborhood renewal and to extend the planning and design phases.

Finally, this study find that socio-demographic indicators have a very important impact on residents’ social capital. For older residents who have lived there for a long time, government workers should make an effort to communicate more with them. This kind of residents usually have a high level of relational social capital. Moreover, residents who have lived in the neighborhood for a long time have a wider social network. Therefore for such residents will play a crucial role in promoting the renewal project.

### 5.3. Limitations and future research

As with any research, this study has some limitations. First, this study suggested that the effect of multi-level social capital in neighborhood renewal should be examined in future research. As proposed by Li et al. (2022) social capital can be classified into resident level and neighborhood level. In this study, the limitation of the data structure did not allow for a multi-level analysis at resident-level and neighborhood-level. Therefore, we suggest using hierarchical linear modeling (HLM) or multilevel structural equation modelling (MLSEM) for assessing the variables in future research. Second, the timing of renewal (currently undergoing renewal, about to begin renewal, or had completed renewal) can be considered in future research. In the process of distributing the questionnaire, the authors of this study found that residents of neighborhoods with different renewal statuses had different attitudes toward the questionnaire. Residents who are going through renewal did not trust authors. Therefore, this study found it difficult to obtain questionnaires from residents of neighborhoods that were undergoing renewal. Therefore, we suggest that future studies could perform multiple group analyses if resident data are available at different times.

## 6. Conclusion

Since residents can be considered the ultimate stakeholders in neighborhood renewal, their social system plays a key role in the smooth implementation of such renewal projects. Building on social capital theory, SEM and MIMIC approaches were used to develop a theoretical framework of the multidimensional social capital of residents in neighborhood renewal in China. The findings confirmed the impact of SSC on both RSC and CSC. We also confirmed the mediating role of RSC between SSC and CSC. As for sociodemographic characteristics, we tested the effects of differences in gender, age, length of residence, and income. Based on our findings, local governments and managers in related fields can develop interventions to improve the performance of neighborhood renewal.

## Data availability statement

The raw data supporting the conclusions of this article will be made available by the authors, without undue reservation.

## Author contributions

RH and XF: conceptualization and data curation. RH: methodology and writing – original draft. FX and WL: resources. WL: supervision. FX, XF, and WL: writing – review and editing. All authors contributed to the article and approved the submitted version.

## Conflict of interest

The authors declare that the research was conducted in the absence of any commercial or financial relationships that could be construed as a potential conflict of interest.

## Publisher’s note

All claims expressed in this article are solely those of the authors and do not necessarily represent those of their affiliated organizations, or those of the publisher, the editors and the reviewers. Any product that may be evaluated in this article, or claim that may be made by its manufacturer, is not guaranteed or endorsed by the publisher.
